# Our Health Counts Toronto: Commercial tobacco use among Indigenous peoples in Toronto

**DOI:** 10.17269/s41997-024-00975-6

**Published:** 2024-11-07

**Authors:** Raglan Maddox, Kristen O’Brien, Chloé G. Xavier, Sara Wolfe, Cheryllee Bourgeois, Janet Smylie

**Affiliations:** 1 Bagumani (Modewa) Clan, Papua New Guinea, Australian National University (ANU), Yardhura Walani, National Centre for Epidemiology and Population Health, Canberra, ACT Australia; 2 Well Living House, Li Ka Shing Knowledge Institute, Unity Health Toronto - St. Michael’s Hospital, Toronto, ON Canada; 3 Seventh Generation Midwives Toronto, Toronto, ON Canada; 4 Dalla Lana School of Public Health and Department of Family and Community Medicine, Faculty of Medicine, University of Toronto, Toronto, ON Canada

**Keywords:** Tobacco use, Smoking, Urban Indigenous health, First Nations, Inuit, Métis, Respondent-driven sampling, Community-based research, Colonialism, Usage de tabac, Fumer, Santé des Autochtones en milieu urbain, Premières Nations, Inuits, Métis, Échantillonnage en fonction des répondants, Recherche basée sur la communauté, Colonialisme

## Abstract

**Objective:**

Fueled by the commercial tobacco industry, commercial tobacco use continues to be the leading preventable cause of premature death in Canada, with opportunities to improve health outcomes. The objective of this research was to work with Indigenous partners to generate Indigenous population prevalence estimates of commercial tobacco use in Toronto, and examine the association between smoking and sociodemographic, cultural, resiliency, and social variables.

**Methods:**

Respondent-driven sampling (RDS) was used to generate prevalence estimates of commercial tobacco use and potentially associated sociodemographic, cultural, resiliency, and social connection variables for Indigenous adults living in Toronto. Statistical analysis examined associations between smoking and variables theorized to be predictors of tobacco use.

**Results:**

The findings indicated that 36.3% (95%CI 28.2–44.5) of the Indigenous population in Toronto do not smoke, and 63.6% (95%CI 55.5–71.7) reported smoking. Univariate analysis of demographic, social, and cultural variables found age and employment to be statistically significantly different between adults who smoked and adults who did not smoke. Indigenous adults who were above the before-tax low-income cut-off (LICO) were more likely to smoke compared to those who were below the before-tax LICO. Indigenous adults who completed high school were more likely to smoke compared to those who did not complete high school, similarly to those who were unemployed compared to those who were employed. However, those who were not in the labour force (student or retired) were less likely to smoke compared to those who were employed. These effects remained after adjustment for age, gender, and LICO. Indigenous adults with stable housing were 20% less likely to smoke compared to those experiencing homelessness. Adults who had at least one close friend or family member to confide in were more likely to smoke compared to those who did not have any close friends or family members. Indigenous adults were more likely to smoke if they participated in Indigenous ceremony compared to those who did not participate.

**Conclusion:**

The Indigenous population in Toronto continues to experience smoking prevalence nearly four times greater than that in the general population. This highlights the need for accurate population data to inform programs and policies and address the social determinants of health.

## Résumé

**Objectif:**

Alimentée par l’industrie du tabac, la consommation de tabac commercial demeure la principale cause évitable de décès prématurés au Canada, ce qui présente une possibilité d’améliorer les résultats cliniques. En collaboration avec des partenaires autochtones, nous avons cherché à estimer la prévalence de la consommation de tabac commercial dans la population autochtone de Toronto et à étudier l’association entre le tabagisme et diverses variables sociodémographiques, culturelles, sociales et de résilience.

**Méthode:**

Nous avons fait appel à l’échantillonnage en fonction des répondants (EFR) pour produire des estimations de la prévalence de la consommation de tabac commercial et d’éventuelles variables connexes (sociodémographiques, culturelles, de liens sociaux et de résilience) chez les adultes autochtones vivant à Toronto. Au moyen d’une analyse statistique, nous avons étudié les associations entre le tabagisme et les variables susceptibles de prédire la consommation de tabac.

**Résultats:**

Selon nos constats, une proportion de 36,3% (IC de 95% 28,2‒44,5) de la population autochtone de Toronto ne fumait pas, et 63,6% (IC de 95% 55,5‒71,7) fumait. L’analyse univariée des variables démographiques, sociales et culturelles a montré que l’âge et l’emploi sont deux variables statistiquement significatives pour expliquer les différences entre les adultes fumeurs et non-fumeurs. Les adultes autochtones se situant au-dessus du seuil de faible revenu (SFR) avant impôt étaient plus susceptibles de fumer que ceux qui se situaient sous le SFR avant impôt. Les adultes autochtones ayant terminé leurs études secondaires étaient plus susceptibles de fumer que ceux qui n’avaient pas terminé leurs études secondaires; de même, les adultes autochtones sans emploi étaient plus susceptibles de fumer que ceux qui avaient un emploi. Cependant, les adultes autochtones qui ne faisaient pas partie de la population active (aux études, à la retraite) étaient moins susceptibles de fumer que ceux qui avaient un emploi. Ces effets persistaient même en tenant compte de l’âge, du sexe et du SFR. Les adultes autochtones ayant un logement stable étaient moins susceptibles de fumer, dans une proportion de 20%, que ceux qui étaient en situation d’itinérance. Les adultes ayant au moins un ami ou parent proche à qui se confier étaient plus susceptibles de fumer que ceux qui n’avaient ni amis, ni parents proches. Les adultes autochtones qui prenaient part à des cérémonies autochtones étaient plus susceptibles de fumer que ceux qui ne participaient pas à ces cérémonies.

**Conclusion:**

À Toronto, la prévalence du tabagisme demeure quatre fois plus importante dans la population autochtone que dans la population générale. Cela souligne le besoin de disposer de données démographiques exactes pour éclairer les programmes et les politiques et pour aborder les déterminants sociaux de la santé.

## Introduction

The colonization of Turtle Island^1^ altered and continues to alter cultures, languages, and traditions of the original inhabitants of this land. This included impacting the relationship with the tobacco plant and how tobacco is used and discussed. While some Indigenous peoples have traditionally used tobacco for ceremonial or sacred purposes, including but not limited to gifting, prayer, and communicating with the Spirit World and the Creator (National Native Network, 2018; Nez Henderson et al., 2022), it is increasingly recognized that the use of commercial tobacco is a major factor in Indigenous health outcomes (Carson, 2014; National Native Network, 2018; World Health Organization, 2003). In Canada, ceremonial tobacco use and ceremonial practices were more broadly illegal under the Indian Act of 1885 and its associated amendments. However, tobacco was enthusiastically appropriated and commercialized, with commercial tobacco legal and permitted through colonial markets in an abundant supply (Dussault & Erasmus, 1996), actively contributing to the systematic promotion of commercial tobacco use and placing a high value on the tobacco plant (Truth and Reconciliation Commission of Canada, 2015a). “Commercial tobacco use” in this paper refers to non-ceremonial use of tobacco products made of the tobacco leaf and manufactured to be used for smoking, sucking, chewing, or snuffing. We humbly acknowledge and respect that Indigenous peoples are diverse and constitute many nations, language groups, and cultures, including relationships with tobacco, across Canada.

Across Canada, cigarette smoking kills 45,000 Canadians each year and commercial tobacco use continues to be the leading preventable cause of premature death (Health Canada, 2018). There are also high rates of commercial tobacco use among First Nations (40%), Inuit (49%), and Métis (37%) peoples (Indigenous peoples) (Health Canada, 2018). In addition to the prevalence of commercial tobacco use among Indigenous peoples, evidence suggests that coloniality has also manifested in a double standard with respect to quality of statistics for Indigenous populations in Canada (Health Canada, 2018; Maddox et al., 2018; Smylie & Firestone, 2015). This is predominately due to data sources that are often biased, are non-random samples, and/or have high levels of non-response or misclassification (Smylie & Firestone, 2015). This results in systematically excluding and/or misclassifying Indigenous peoples, consequently undermining our understanding of commercial tobacco use among the Indigenous population, virtually masking Indigenous smoking prevalence and misleading commercial tobacco reduction programs and policies. As a result, we currently have limited understanding of commercial tobacco use, such as accurate baseline data as well as more detailed and nuanced understandings of types of commercial tobacco use (not just cigarettes), frequency of use (smoking prevalence can be defined in varied ways), quitting behaviours, and commercial tobacco–related health harms and mortality (Gionet & Roshanafshar, 2013; Smylie & Anderson, 2006; Smylie & Firestone, 2015).

The reasons for the disproportionately high rates of commercial tobacco use among Indigenous peoples are complex and multifactorial (Carson, 2014; Carson et al., 2012a, b; Nez Henderson et al., 2022; Thomas et al., 2015; Waa, 2012). Research indicates that the drivers associated with taking up and sustaining commercial tobacco use are similar for Indigenous and non-Indigenous peoples. However, as a result of the ongoing impacts of colonization and the colonial mechanics that manufacture the presence of these drivers sustaining tobacco use, such as exclusion from the economy and the education system, Indigenous peoples more commonly experience these drivers of commercial tobacco use (Thomas et al., 2015; Waa, 2012). Indigenous status is also commonly and mistakenly used as an explanatory variable for high prevalence of commercial tobacco use (Maddox et al., 2018; Pearson et al., 2021). However, Indigeneity is a proxy marker for a constellation of socioeconomic and other contributing factors (Chamberlain et al., 2017; Thomas et al., 2015; Waa, 2012). The roots of such causes can be found in historic and current governmental programs and policies that disrupt Indigenous societies. This includes colonization and the associated impacts, such as ongoing experiences of racism—acute, attitudinal, and systemic racism in the interface with non-Indigenous systems, institutions, and structures, such as the health care system and penal system—and added stressors, such as the intergenerational erosion of social structures, economies, and resources (Chamberlain et al., 2017; Truth and Reconciliation Commission of Canada, 2015a).

It is vital that commercial tobacco reduction programs are based on Indigenous ways of knowing, doing, and being, in which health and well-being encompass physical health along with environmental, spiritual, and cultural well-being. This needs to consider historical, cultural, and social factors, including the ongoing impacts of colonization. In order to accelerate declines in commercial tobacco prevalence, the overall approach to reducing tobacco use needs to be underpinned by proportionate universalism and consistent with the United Nations Declaration on the Rights of Indigenous Peoples (UNDRIP). In addition, Indigenous-specific commercial tobacco control evidence needs to be culturally appropriate and tailored to community needs (i.e. targeted campaigns and programming) (Chamberlain, 2017), holistic in addressing the social determinants of health, and comprehensive and multi-faceted with long-term funding.

This is consistent with the Indigenous tobacco control evidence and the World Health Organization (WHO) Framework Convention on Tobacco Control (FCTC) (Carson et al., 2012a, b; Chamberlain et al., 2017; Minichiello et al., 2016; World Health Organization, 2003). Canada has been a signatory to the FCTC since it came into force in 2005, recognizing the commercial tobacco–related health harms experienced by Indigenous peoples (World Health Organization, 2003). The health harms of commercial tobacco use are well established as we have known about the harms of commercial tobacco use for over 70 years (United States Surgeon General’s Advisory Committee on Smoking, 1964). It is critically important that Indigenous peoples can develop, implement, and evaluate socially and culturally appropriate sustainable tobacco reduction programs and policies, informed by accurate population prevalence estimates of commercial tobacco use.

The primary objective of this paper was to work in partnership with Indigenous community health service partners to accurately generate population prevalence estimates of commercial tobacco use within the Indigenous population in Toronto and examine the association between smoking and specific sociodemographic, cultural, resiliency, and social connection variables known or hypothesized to be predictors of commercial tobacco use.

## Methods

### Our Health Counts Toronto survey

The Our Health Counts (OHC) Toronto Respectful Health Survey was developed to create a comprehensive database of individual, family, and community health and well-being measures among the Indigenous community in Toronto. Since 2010, OHC has been successfully administered in six urban centres in Ontario. The information from these surveys has served an important role to better understand health and well-being needs of Indigenous peoples in a number of urban settings in Ontario, Canada.

### Indigenous community partnerships

As detailed throughout the current CJPH Special Issue as well as in Rotondi et al. (2017) and Firestone et al. (2018), OHC Toronto used Indigenous community–driven processes to generate a comprehensive health information platform to understand and address critical gaps in urban Indigenous health and commercial tobacco use. This study was co-led by the Seventh Generation Midwives Toronto (SGMT) and the Well Living House Action Research Centre for Indigenous Infant, Child and Family Health and Wellbeing (Well Living House) at St. Michael’s Hospital. SGMT is a midwifery clinic in Toronto that specializes in providing care for Indigenous mothers and their babies. Given the traditional role of midwives as knowledge keepers of birth stories and family information, SGMT was a fitting organizational community custodian for Indigenous health data in Toronto (Smylie et al., 2012).

### Study site

The City of Toronto is located in southern Ontario and is situated on the traditional territory of many Indigenous nations, including the Mississaugas of the Credit, the Anishnabeg, the Chippewa, the Haudenosaunee, and the Wendat peoples, and is covered by Treaty 13 with the Mississaugas of the Credit. Toronto is the largest urban centre in Canada, with a population of 2.7 million people (Statistics Canada, 2016). According to the 2016 Canadian Census, Indigenous peoples make up less than 1% of the population of Toronto (Statistics Canada, 2016), although this has been shown to be a substantial underestimate (Rotondi et al., 2017). Surveys were conducted by Indigenous interviewers at three Indigenous or allied organizations located in different regions of downtown Toronto. Home visits were also available if potential participants were unable to access the survey locations.

### Eligibility

Individuals aged 15 years or older who self-identified as Indigenous (First Nations, Inuit, or Métis) and resided, worked, or used health services in Toronto were eligible to participate.

### Respondent-driven sampling

Respondent-driven sampling (RDS), a statistical technique used to estimate populations of hard-to-reach groups, was implemented as the sampling framework to address the aforementioned challenges which systematically exclude and/or misclassify Indigenous peoples (Firestone et al., 2014; Heckathorn, 1997, 2002; Rotondi et al., 2017). This built on the strong social networks among the Indigenous community in Toronto (Rotondi et al., 2017). To begin the recruitment process, 20 individuals from the Indigenous community in Toronto were chosen as “seeds”. Seeds were selected based on diverse characteristics in an attempt to reach various subgroups within the population.

The seeds completed the survey and were provided training on how to recruit new participants. Each seed was given three to five coupons to distribute to eligible friends, family, or acquaintances within their social network. New recruits brought their coupon to one of the study locations and upon completion of the survey were provided coupons to distribute within their social network. The referral pattern continued until equilibrium was reached. Study participants were provided a $20 honorarium for completing the survey, an additional $10 for each child survey they completed for a child in their care, and an additional $10 for each successful recruit who participated.

### Primary outcome and covariates

The primary outcome, smoking status, was constructed based on the survey question asking “Do you currently smoke cigarettes?” The relationships between smoking status and specific sociodemographic, cultural, resiliency, and social connection variables known or hypothesized to be predictors of commercial tobacco use were examined. Sociodemographic variables directly evidenced to impact commercial tobacco use and examined included age, gender, before-tax low-income cut-off, education, employment, housing, overcrowding, and mobility (moves in the past year). Potential factors associated with commercial tobacco use were identified from reviews of Indigenous-specific literature (Carson, 2014; Carson et al., 2012a, b; Chamberlain et al., 2017; Colonna et al., 2020; Minichiello et al., 2016). We also sought community input, including input from SGMT, to ensure local community relevance. This highlighted the need to include potential associations with two-spirit identity and commercial tobacco, helping to consider the unique experiences of the community. Due to growing evidence that Indigenous cultures can influence health and well-being (Bourke et al., 2018), a range of cultural variables were hypothesized to be protective towards commercial tobacco use. We explored the ability to speak an Indigenous language, activity within an Indigenous organization, sense of cultural belonging, participation in Indigenous ceremony, and use of Indigenous medicines or practices. Discrimination and experiences of trauma are documented to impact commercial tobacco use, highlighted through racialized disparities (Williams & Mohammed, 2009). As a result, we explored measures of resilience, including child protection agency involvement as a child, surviving residential school, or having a missing friend or family member. Social connection was measured by the number of close friends or family members with whom the participant felt comfortable sharing what was on their mind.

### Statistical analysis

To adjust for the RDS sampling framework, RDS-II weights were constructed in R using the RDS package (V.0.7.7) and used for all analyses. Chi-square tests were used to assess the association of predictors and current smoking status. Three models were constructed to assess relationships between individual predictors and the current smoking status while controlling for demographic factors. Model 1 was unadjusted/bivariate, model 2 controls for age and gender, and model 3 controls for age, gender, and before-tax low-income cut-off (LICO). Before-tax LICOs are income thresholds developed by Statistics Canada to identify households potentially living in poverty. These thresholds assess whether a family is likely to spend a significantly larger portion of its income on basic necessities—such as food, shelter, and clothing—compared to the average household. LICOs vary by household size and geographic location, and families earning below these thresholds are considered to be in low income (Statistics Canada, 2024). Generalized linear mixed models were used to account for the correlation and clustering among recruits from the same seed. Odds ratios and 95% confidence intervals were constructed for all models. Statistical analyses were conducted using SAS 9.4 (SAS Institute Inc., Cary, NC, USA).

### Ethics

This Indigenous-led community research project included Indigenous peoples at all levels of the research and across all stages of the research process, from pre-conception to dissemination of results and findings while ensuring the ethical conduct of research involving Indigenous peoples as detailed in the current CJPH Special Issue. This included ongoing review and input through OHC Toronto Reference Group and core partner meetings, as well as general guidance and support from the Well Living House Counsel of Grandparents.

Ethics approval was also obtained from St. Michael’s Hospital (REB# 14–083).

## Results

### Sample/participants

A total of 915 Indigenous adults completed surveys between April 2015 and March 2016. Of those surveyed, 844 Indigenous adults lived in the City of Toronto, were not seeds, and provided a response for the smoking status question (Table 1).

**Table 1:** Descriptive statistics of people who smoke and people who do not smoke

Variable	Level	Total (*n* = 884)				Do you currently smoke cigarettes?								*p*-value
						Yes (*n* = 605)				No (*n* = 279)				
		Frequency	RDS %	Lower 95%CI	Upper 95%CI	Frequency	RDS %	Lower 95%CI	Upper 95%CI	Frequency	RDS %	Lower 95%CI	Upper 95%CI	
Do you currently smoke cigarettes?						605	63.7	55.5	71.8	279	36.3	28.2	44.5	
Indigenous identity	First Nations and Métis	8	0.5	0.0	1.0	7	0.7	0.0	1.5	1	0.0	0.0	0.1	*
	First Nations	784	85.9	80.2	91.7	528	84.4	76.7	92.2	256	88.6	80.6	96.5	
	Métis	80	13.0	7.3	18.7	59	13.9	6.2	21.6	21	11.4	3.4	19.3	
	Inuit	9	0.3	0.1	0.6	8	0.5	0.1	0.9	1	0.0	0.0	0.1	
	Other^a^	3	0.3	0.0	0.7	3	0.4	0.0	1.1	0				
Age group	15 to 34	299	40.7	32.8	48.7	210	32.5	25.0	39.9	89	55.2	40.7	69.7	0.0016
	35 to 54	404	45.3	37.2	53.3	296	54.0	45.3	62.8	108	29.9	16.9	42.9	
	55 and over	181	14.0	10.1	17.9	99	13.5	8.7	18.3	82	14.9	8.3	21.5	
Two-Spirit identity	Yes	185	22.7	16.3	29.0	140	26.4	17.9	34.9	45	16.2	8.1	24.2	0.0854
	No	699	77.3	71.0	83.7	465	73.6	65.1	82.1	234	83.8	75.8	91.9	
Gender	Women	450	48.4	40.3	56.4	280	44.5	35.4	53.6	170	55.0	39.7	70.4	0.2495
	Men	417	51.6	43.6	59.7	314	55.5	46.4	64.6	103	45.0	29.6	60.3	
Education	Less than high school	386	50.9	42.9	58.9	274	49.6	40.5	58.7	112	53.2	38.3	68.2	0.3949
	High school	168	17.3	11.7	22.9	132	20.3	12.6	27.9	36	12.1	5.3	18.9	
	More than high school	328	31.8	24.4	39.2	199	30.1	21.7	38.6	129	34.7	20.7	48.7	
Employment	Employed	254	17.2	13.0	21.4	167	16.5	11.9	21.1	87	18.5	10.1	26.8	< 0.0001
	Unemployed	467	64.4	56.9	72.0	351	74.6	68.5	80.8	116	46.9	31.6	62.2	
	Not in labour force	129	18.3	11.2	25.4	62	8.9	5.2	12.6	67	34.6	18.8	50.4	
Before-tax LICO	Above before-tax LICO	171	12.3	9.0	15.6	120	13.0	9.0	17.0	51	11.1	5.4	16.8	0.601
	Below before-tax LICO	702	87.7	84.4	91.0	477	87.0	83.0	91.0	225	88.9	83.2	94.6	
Housing type	Stable housing	635	63.6	55.1	72.0	408	59.2	49.4	68.9	227	71.0	55.7	86.4	0.3911
	Precarious housing + institution	72	8.5	5.4	11.6	54	9.3	5.5	13.1	18	7.3	2.1	12.5	
	Homeless	152	27.9	19.3	36.5	122	31.6	21.4	41.7	30	21.7	6.1	37.2	
Household crowding	Overcrowded	68	13.3	6.2	20.5	41	14.8	4.2	25.4	27	11.1	3.9	18.3	0.5544
	Not overcrowded	612	86.7	79.5	93.8	401	85.2	74.6	95.8	211	88.9	81.7	96.1	
Moves in the past year	No moves	520	47.6	39.6	55.6	339	44.2	35.5	52.9	181	53.4	38.1	68.8	0.2994
	1 or more moves	344	52.4	44.4	60.4	251	55.8	47.1	64.5	93	46.6	31.2	61.9	
Speaks an Indigenous language	Yes	425	41.5	33.5	49.5	269	42.0	32.7	51.3	156	40.6	25.7	55.5	0.8712
	No	459	58.5	50.5	66.5	336	58.0	48.7	67.3	123	59.4	44.5	74.3	
About how many close friends and close relatives do you have, that is, people you feel at ease with and can talk to about what is on your mind?	1 or more	793	90.4	87.1	93.7	540	92.0	88.6	95.5	253	87.5	80.6	94.3	0.2105
	None	91	9.6	6.3	12.9	65	8.0	4.5	11.4	26	12.5	5.7	19.4	
Was a child protection agency ever involved in your care when you were a child?	Yes	408	46.5	38.6	54.5	283	47.9	38.7	57.0	125	44.2	29.4	58.9	0.6729
	No	468	53.5	45.5	61.4	317	52.1	43.0	61.3	151	55.8	41.1	70.6	
Were you ever a student at a federal residential school, or a federal industrial school?	Yes	75	11.1	4.6	17.5	43	10.2	2.8	17.6	32	12.6	0.7	24.6	0.7207
	No	803	88.9	82.5	95.4	557	89.8	82.4	97.2	246	87.4	75.4	99.3	
Has a close friend or family member ever gone missing?	Yes	284	25.8	19.4	32.2	188	25.0	18.2	31.9	96	27.1	14.2	40.0	0.775
	No	590	74.2	67.8	80.6	410	75.0	68.1	81.8	180	72.9	60.0	85.8	
I am active in organizations or social groups that include mostly Indigenous people	Strongly agree/agree	647	64.1	55.9	72.3	435	66.9	58.2	75.6	212	59.3	43.3	75.3	0.3961
	Disagree/strongly disagree	227	35.9	27.7	44.1	160	33.1	24.4	41.8	67	40.7	24.7	56.7	
I have a clear sense of my cultural background as an Indigenous person and what that means to me	Strongly agree/agree	735	79.3	72.4	86.2	488	77.4	69.4	85.5	247	82.5	69.7	95.3	0.4776
	Disagree/strongly disagree	138	20.7	13.8	27.6	107	22.6	14.5	30.6	31	31.0	17.5	4.7	0.3065
Do you participate in traditional Indigenous ceremony (i.e. smudge, sweat lodge, fast, healing Qulliq or Kudlik lamp lighting ceremony)?	Yes	670	64.8	56.9	72.7	463	69.6	61.2	78.0	207	56.5	41.2	71.9	0.127
	No	214	35.2	27.3	43.1	142	30.4	22.0	38.8	72	43.5	28.1	58.8	
Do you use traditional Indigenous medicines or practices to maintain your health and well-being?	Yes	541	48.4	40.5	56.3	362	47.7	38.8	56.6	179	49.7	34.5	64.8	0.8276
	No	342	51.6	43.7	59.5	243	52.3	43.4	61.2	99	50.3	35.2	65.5	

^a^One non-Indigenous participant, with an Indigenous child; and two Indigenous participants from countries other than Canada

### Descriptive characteristics of smokers and non-smokers

Thirty percent (30%) of Indigenous participants reported that they did not currently smoke, while 70% (*n* = 617) reported they currently smoke cigarettes. RDS-adjusted population estimates indicated that of the Indigenous population in Toronto, 36.3% (95%CI 28.2–44.5) did not smoke and 63.7% (95%CI 55.5–71.7) currently smoke cigarettes.

Among various demographic, social, and cultural factors examined through univariate analysis (Table 2), only age and employment differed between those who smoked cigarettes and those who did not. Indigenous adults who did not smoke cigarettes tended to be younger (15 to 34 years of age) and employed or not in the labour force (student or retired) versus unemployed. No differences were noted in the descriptive analysis between those who did and those who did not smoke with respect to gender, education, income, housing, mobility, historical and intergenerational trauma, importance of Indigenous identity, participation in ceremony, or use of medicines.

**Table 2:** Unadjusted and adjusted multivariable models modeling

Variable	Level	Unadjusted model			Adjusted models controlling for age and gender			Adjusted models controlling for age, gender, and LICO		
		Odds ratio (unadjusted)	Lower 95%CI	Upper 95%CI	Odds ratio adjusted by age and gender	Lower 95%CI	Upper 95%CI	Odds ratio adjusted by age, gender, and LICO	Lower 95%CI	Upper 95%CI
Two-Spirit	Yes	2.040	0.558	7.462	2.214	0.732	6.693	2.271	0.760	6.787
	No	1			1			1		
Education	Less than high school	1			1			1		
	High school	1.915	1.410	2.599	1.743	1.319	2.303	1.749	1.223	2.502
	More than high school	0.933	0.520	1.673	0.715	0.346	1.476	0.703	0.313	1.583
Employment	Employed	1			1			1		
	Unemployed	1.954	0.974	3.923	1.600	0.881	2.905	1.698	1.022	2.821
	Not in labour force	0.272	0.156	0.473	0.352	0.209	0.592	0.352	0.205	0.604
Housing	Stable housing	0.61	0.473	0.786	0.773	0.614	0.972	0.753	0.602	0.942
	Precarious housing + institution	0.888	0.742	1.063	0.865	0.607	1.234	0.826	0.578	1.18
	Homeless	1			1			1		
Overcrowding	Yes	1.271	0.702	2.300	1.209	0.821	1.779	1.020	0.597	1.746
	No	1			1			1		
Moves in the past year	No moves	1			1			1		
	1 or more moves	1.508	1.082	2.103	1.770	1.211	2.586	1.758	1.129	2.736
Speak Indigenous language	Yes	1.027	0.784	1.346	0.883	0.661	1.178	0.875	0.646	1.185
	No	1			1			1		
About how many close friends and close relatives do you have, that is, people you feel at ease with and can talk to about what is on your mind?	1 or more	1.649	1.081	2.514	2.259	1.686	3.025	2.258	1.697	3.005
	None	1			1			1		
Was a child protection agency ever involved in your care when you were a child?	Yes	1.143	0.840	1.555	1.016	0.817	1.264	1.001	0.813	1.232
	No	1			1			1		
Were you ever a student at a federal residential school, or a federal industrial school?	Yes	0.776	0.247	2.440	0.604	0.187	1.946	0.602	0.178	2.031
	No	1			1			1		
Has a close friend or family member ever gone missing?	Yes	0.91	0.511	1.618	0.896	0.477	1.683	0.881	0.467	1.661
	No	1			1			1		
Active in Indigenous organizations	Strongly agree/agree	1.366	0.912	2.048	1.228	0.820	1.84	1.229	0.847	1.783
	Disagree/strongly disagree	1			1			1		
Sense of cultural background	Strongly agree/agree	0.704	0.264	1.872	0.657	0.226	1.907	0.655	0.221	1.940
	Disagree/strongly disagree	1			1			1		
Do you participate in traditional Indigenous ceremony (i.e. smudge, sweat lodge, fast, healing Qulliq or Kudlik lamp lighting ceremony)?	Yes	1.749	1.473	2.076	1.685	1.326	2.141	1.691	1.331	2.147
	No	1			1			1		
Do you use traditional Indigenous medicines or practices to maintain your health and well-being?	Yes	0.963	0.723	1.283	1.008	0.781	1.300	0.982	0.745	1.294
	No	1			1			1		

### Unadjusted and adjusted multivariable results

In the unadjusted (bivariate) models, those who were above the before-tax LICO had 1.43 times the odds (1.02, 2.04) of current smoking compared to those who were below the before-tax LICO and Indigenous adults who completed high school were more likely to smoke compared to Indigenous adults who did not complete high school (OR 1.92, 95%CI 1.41–2.60).

After adjusting for age, gender, and before-tax LICO, Indigenous adults who were unemployed were more likely to smoke compared to those who were employed (OR 1.70, 95%CI 1.02–2.82). However, those who were not in the labour force (student or retired) were less likely to smoke compared to those who were employed (OR 0.27, 95%CI 0.16–0.47). These effects remained after adjustment for age, gender, and LICO. Similarly, those experiencing homelessness were more likely to smoke compared to those who in stable housing (OR 0.61, 95%CI 0.47–0.79) and those who moved at least once in the past year were more likely to smoke compared to those who did not move in the past year (OR 1.51, 95%CI 1.08–2.10).

Adults who had at least one close friend or family member to confide in had 1.65 times the odds of smoking compared to those who did not have any close friends or family members. After adjusting for age, gender, and LICO, the odds increased to 2.26 (1.70, 3.01).

Indigenous adults had 1.75 (1.47, 2.08) times the odds of smoking if they participated in Indigenous ceremony compared to those who did not participate. Adjusting for age, gender, and LICO did not impact the estimated odds ratio.

## Discussion

The Indigenous population in Toronto is one of the largest Indigenous communities in Canada (Rotondi et al., 2017; Statistics Canada, 2013) and in spite of ongoing impacts of colonization, 36.3% of the Indigenous population in Toronto do not smoke. However, 63.7% (approximately 45,227) of Indigenous adults currently smoke, reflecting significant opportunities to reduce commercial tobacco use and consequently, improve health outcomes. We identified a population prevalence of commercial tobacco use that is more than four times higher than that of the general Toronto population (Toronto Public Health, 2022). This highlights the normalization of commercial tobacco use that has been embedded through colonization and the ongoing mechanics of colonization, regardless of sociodemographic, cultural, resiliency, and social connection. Colonization has led to ongoing trauma, stress, racism, and exclusion from education and employment, all of which have been linked to commercial tobacco use (Colonna et al., 2020; Thurber et al., 2021; Truth and Reconciliation Commission of Canada, 2015a; Waa et al., 2019). These findings additionally highlight the extent of commercial tobacco use and the significant opportunity to improve Indigenous health outcomes, stressing the urgent need to resource and support Indigenous-led and Indigenous-driven commercial tobacco reduction programs and policies (Carson, 2014; World Health Organization, 2003).

Indigenous adults who were unemployed were more likely to smoke compared to those who were employed when adjusting for age, gender, and before-tax LICO. However, those who were not in the labour force (student or retired) were less likely to smoke compared to those who were employed (OR 0.27, 95%CI 0.16–0.47). This may be due to the “sick quitter effect”, with high levels of multimorbidity and the normalization of commercial tobacco use resulting in disease onset, changes in health condition leading to cessation, and commercial tobacco-free behaviours. However, the effects remained for those in the labour force and those who were employed while adjusting for age, gender, and LICO; highlighting the fundamentally important contributions of the social determinants of commercial tobacco use. This includes robust growing evidence regarding indicators of socioeconomic status, such as labour force engagement, the impact of Indigenous exclusion from the economy, and the education system, as Indigenous peoples more commonly than non-Indigenous peoples experience the drivers of commercial tobacco use (Thomas et al., 2015; Waa, 2012).

In Canada, ceremonial practices including ceremonial tobacco use were more broadly illegal under the Indian Act of 1885 and its associated amendments (Colonna et al., 2020; Thurber et al., 2021; Truth and Reconciliation Commission of Canada, 2015b; Waa et al., 2019). However, this actively contributed to the promotion of commercial tobacco use among First Nations (status and non-status) and Métis peoples, as commercial tobacco use was not illegal (Boudreau et al., 2016; Flannery et al., 1995; Truth and Reconciliation Commission of Canada, 2015b). As a result, commercial tobacco products were introduced into ceremonial practices as a harmful and unsustainable replacement to sacred tobacco. The restrictions of cultural and ceremonial practices, including use of ceremonial tobacco, were finally lifted in in 1951. Acknowledgement of colonial undermining of the sacred relationship with tobacco is ongoing. This colonial socialization, normalization, and association of commercial tobacco use and ceremony going hand in hand is reflected with Indigenous adults having 1.75 (1.47, 2.08) times the odds of smoking if they participated in Indigenous ceremony compared to those who did not participate (de Leeuw, 2017; Nez Henderson et al., 2022). This highlights the importance of valuing, integrating, and embedding traditional healing and ceremonies into Euro-Western medicine to help decolonize tobacco use and foster commercial tobacco-free norms (de Leeuw, 2017). Further, the socialization of commercial tobacco and its role in relationship reciprocity has been pervasive, acting as a social lubricant (Maddox, 2015) fueled by the commercial tobacco industry, and a mechanism of colonial intrusion on Indigenous ways of visiting: knowing, being, and doing (Maddox, 2015; Waa et al., 2019).

Avoiding smoking initiation and successful smoking cessation are complex behaviours influenced by historical and contemporary systemic factors (Scollo & Winstanley, 2019). Reducing commercial tobacco use at the population level requires a suite of comprehensive approaches to halt initiation and promote cessation (Chamberlain et al., 2017; World Health Organization, 2003), as well as addressing structural and systemic barriers to reducing commercial tobacco use (Carson, 2014; Carson et al., 2012a, b; Lovett et al., 2017). In addition to Indigenous-specific commercial tobacco control programming, reducing the underlying social and economic exclusionary factors is crucial to reducing commercial tobacco use and the associated morbidity and mortality.

Indigenous adults who did not smoke cigarettes tended to be younger and there was high prevalence among men and women, unemployed, and those without secure housing—similar to other Indigenous populations and highlighting a number of common ongoing colonial impacts (Colonna et al., 2020; Thurber et al., 2021).

Commercial tobacco use is common and accelerating reductions is urgently required. It is well established that tobacco use has significant impacts on health, and there are vast benefits to being smoke free. Quitting smoking, or never starting to smoke, are complex behaviours and as a result, evidence is required to better understand “what works” in tobacco control to accelerate reductions in tobacco use among Indigenous peoples in Toronto, Ontario. Tobacco control programs and policies can help people and communities understand the health harms and impacts of tobacco use, promoting smoke-free norms. There is a wide range of tobacco control approaches that are recommended to effectively work with and target individuals and communities to promote cessation and discourage uptake.

### Strengths and limitations

The Indigenous governance and meaningful engagement, including input and participation from Indigenous peoples at all stages of the research process, adding culturally relevant insights and emphasizing the right to be counted was a strength of this study. It is important that this study used RDS sampling, since there is no existing representative, inclusive sampling frame for First Nations, Inuit, and Métis peoples living in cities in Canada. RDS requires larger sample sizes than random sampling methods, which impacted study precision for some analysis and precluded creation of First Nations-, Inuit-, and Métis-specific sub-analyses. This research provides foundational baseline cross-sectional data. However, this is not generalizable and precludes our ability to truly understand complex causal pathways and the intersectionality present and lacks a detailed understanding of the types and frequency of commercial tobacco use, quitting behaviours, and Indigenous-specific commercial tobacco–related health morbidity and mortality data. Finally, we examined a substantial number of independent variables (Table 2) which increases the chance that associations may be identified by chance. The variables were identified from reviews of Indigenous-specific literature, with input, discussion, and prioritization by the local community, highlighting the importance of the Indigenous governance and meaningful engagement to help ensure local relevance and scientific excellence.

## Conclusion

There is an urgent need for recent and accurate Indigenous population health data to shape and inform appropriate commercial tobacco reduction programs and policies, assisting to reduce a completely preventable cause of morbidity and mortality. While Canada is a signatory to the FCTC which recognizes the disproportionate harm caused by tobacco use and the need to engage with Indigenous peoples in planning, delivery, and evaluation of commercial tobacco reduction programs and policies, there is substantial work to be undertaken. This includes Indigenous-driven commercial tobacco reduction programs and policies informed by accurate prevalence data, as well as addressing structural and systemic factors that influence commercial tobacco use. The Indigenous population in Toronto continues to experience high commercial tobacco prevalence, highlighting a double standard with respect to commercial tobacco reduction programs, policies, and data collection systems, with significant room for improvement.

## Contributions to knowledge

What does this study add to existing knowledge?

- This study provides the first accurate population-level estimates of Indigenous peoples and commercial tobacco use in Toronto, indicating that 36.3% of the population did not smoke and 63.6% reported smoking.
- The study highlights influential factors, including employment, housing, racism, and colonization, emphasizing the need for culturally appropriate tobacco control programs and policies.

What are the key implications for public health interventions, practice, or policy?

- The study highlights the importance of community-driven tobacco control programs and policies, addressing social determinants of smoking, including housing, employment, racism, and colonization.
- Policies should support Indigenous communities in developing, implementing, and evaluating strategies to reduce commercial tobacco use and its impact.

## Data Availability

Data are owned by the Indigenous community partners.

## Footnotes

1 Turtle Island is frequently known as North America.
